# Enhancing genomic selection by fitting large-effect SNPs as fixed effects and a genotype-by-environment effect using a maize BC_1_F_3:4_ population

**DOI:** 10.1371/journal.pone.0223898

**Published:** 2019-10-17

**Authors:** Dongdong Li, Zhenxiang Xu, Riliang Gu, Pingxi Wang, Demar Lyle, Jialiang Xu, Hongwei Zhang, Guogying Wang

**Affiliations:** 1 National Key Facility for Crop Gene Resources and Genetic Improvement, Institute of Crop Sciences, Chinese Academy of Agricultural Sciences, Beijing, P. R. China; 2 Center for Seed Science and Technology, College of Agronomy and Biotechnology, China Agricultural University, Beijing, P. R. China; Sichuan Agricultural University at Chengdu, CHINA

## Abstract

The popularity of genomic selection (GS) has increased owing to its prospects in commercial breeding. It is necessary to enhance GS to increase its efficiency. In this study, a maize BC_1_F_3:4_ population, consisting of 481 families, was evaluated for days to anthesis in four environments, and genotyped with DNA chips including 55,000 single nucleotide polymorphisms (SNPs). This population was used to investigate whether GS could be enhanced by borrowing information from the genetic basis and genotype-by-environment (G × E) interaction. The results showed that: 1) fitting the top four large-effect SNPs as fixed effects could increase prediction accuracy, including three minor-effect SNPs explaining less than 10% phenotypic variance; 2) the increase of prediction accuracy when fitting large-effect SNPs as fixed effects was related to the decrease of genetic variance; 3) generally, the GS model fitting large-effect SNPs as fixed effects and G × E component enhanced GS. Therefore, we propose fitting large-effect markers as fixed effects and G × E effect for crop breeding projects in order to obtain accurately predicted phenotypic data and conduct efficient selection of desired plants.

## Introduction

Plant quantitative genetics is a burgeoning field, enabling the identification of a great number of quantitative trait locus/loci (QTL) and genes in crops. The QTL or gene information (including its position and effect) should be summarized and transferred to molecular markers to better serve crop breeding [1, 2]. The conventional use of QTL or gene information in plant breeding typically involves of marker-assisted selection (MAS), which requires the identification of significant QTL and selection of desired plants in advanced populations [3]. In the MAS method, the target QTL are usually major QTL, and minor QTL are often not detected due to the probability of false negative, thus the QTL information is not fully exploited [3]. Genomic selection (GS), which was introduced in animals and later applied to crop genetic research, could make use of minor-effect QTL for the improvement of target traits [4, 5].

GS entails the prediction of genomic estimated breeding values (GEBVs) of a validation population based on a training population, for which both phenotypic and genotypic data are available [4]. Factors influencing the prediction accuracy (PA) of GS should be considered and optimally controlled to accurately estimate GEBVs. These factors include, but are not limited to, population size, marker density, heritability, linkage disequilibrium, the genetic architecture of the target traits (QTL number, QTL effects, and QTL interactions), genetic relatedness between the training and validation populations, and GS models [6–8]. Some factors are difficult to modify when both genotypic and phenotypic data are available, whereas others can be optimized using statistical approaches. There might be differences in PAs among GS models, including ridge regression best linear unbiased prediction (rrBLUP), genomic best linear unbiased prediction (GBLUP), or the Bayesian alphabet [5, 9, 10]. However, the selection of an optimal GS model has proven difficult, as there is no clear indication of which model improves PA in all cases [9, 11, 12].

Examining of the components of GS models may provide insight into improving the PAs of GS models. In an rrBLUP model, the linear model is **y = Xβ+Zu+ε**, which is composed of fixed effect **β** and random effect **u**. Modifying the fixed and random effects might improve GS models. For example, a simulation study found that fitting major-effect SNPs as fixed effects could enhance genomic prediction [13, 14]. A study on wheat stem rust resistance found that the PA of a GBLUP model using markers linked to Sr2 (involved in stem rust resistance) as fixed effects was larger than that of an ordinary GBLUP [14]. Generally, it is beneficial to use known genetic or gene information to improve GS models. In maize, it is common to select breeding materials from the offsprings of F_1_ plants [15]. Therefore, it is necessary to confirm the effect of fitting large-effect markers as fixed effects in GS models using a maize biparental population.

The use of additional random variables might be helpful in enhancing the prediction of GS models. As suggested by a study on wheat, the PA of a GS model including a genotype-by-environment (G × E) effect was higher than that of other GS models [16]. In another study on dairy cattle, the PA of a G × E GS model was the higher than other models for predicting metabolic body weight [17]. It is important to study the effect of modeling G × E in maize, which shows adaptations to various environments ranging from tropical to temperate regions. Flowering time is crucial for the adaptation of maize to diverse ecological regions, and is easily influenced by the environment, especially temperature and photoperiod [18, 19]. It was demonstrated that QTL controlling flowering time were closely related to environmental conditions, some QTL showed significant QTL × environmental interaction effects [18, 20]. Therefore, flowering time is suitable for analyzing the contribution of G × E to the PA of GS model.

In this study, 481 maize BC_1_F_3:4_ families were constructed using elite inbred lines. Meanwhile, genotypic and phenotypic data of the 481 families were obtained. The objectives of this study were to investigate whether fitting large-effect SNPs as fixed effects could increase the PAs of GS models, and whether modeling G × E interaction could increase the PAs of GS models. Afterward, we assessed the performance of the GS models fitting large-effect SNPs as fixed effects and G × E interaction. This analysis would provide insight into improving the PAs of GS models using available phenotypic and genotypic data.

## Materials and methods

### Plant materials and phenotyping

A biparental population was constructed using elite inbred lines Zheng58 and PH4CV. Zheng58 is the female parent of Zhengdan958 and PH4CV is the male parent of Xianyu335. Zhengdan958 and Xianyu335 are popular hybrids in China [21, 22]. The F_1_ plants were backcrossed to PH4CV to produce the BC_1_F_1_ seeds. Each BC_1_F_1_ plant was pollinated with bulked pollens collected from at least ten other BC_1_F_1_ plants in the summer of 2014 in Shunyi, Beijing. The offsprings of these BC_1_F_1_ plants were defined as bulk-BC_1_F_2_. In the summer of 2015 in Shunyi, Beijing, forty-three bulk-BC_1_F_2_ families were sown, with each family in a one-row plot. Three plants in each row were self-pollinated to produce the BC_1_F_3_ seeds. In the winter of 2015 in Sanya, Hainan, 481 BC_1_F_3_ plants from three BC_1_F_2_ ears sown and self-pollinated to produce 481 BC_1_F_3:4_ families. The flowchart for the construction of materials used in this study was demonstrated in S1 Fig. The BC_1_F_3:4_ families were sown in Shunyi, Beijing, and Changji, Xinjiang, in the summer of 2016 and 2017, the four environments were identified as 16BJ, 17BJ, 16XJ and 17XJ, respectively. In each environment, the BC_1_F_3:4_ families were planted in a randomized complete design with two replicates. Within each replicate, each family was sown in a one-row plot. The row space was 50cm and the distance between two neighboring plants was 25cm. DA was recorded when 50% of the plants in each plot reached anthesis. The phenotypic data of DA are included in S1 File.

### Phenotype data analysis

The best linear unbiased estimates (BLUEs) of the 481 BC_1_F_3:4_ families were estimated following the model:

$$y_{ijm}=\mu +g_{i}+e_{j}+{ge}_{ij}+\delta_{(j)m}+\varepsilon_{ijm},$$

where *y*_*ijm*_ is the phenotype of the *i*^*th*^ (i = 1,2 …,481) genotype in the *j*^*th*^ (*j* = 1,2,3,4) environment, the *m*^*th*^ (m = 1,2) replicate effect was nested in each environment. μ is the overall mean, *g*_*i*_ is the genotype effect, *e*_*j*_ is the environmental effect, *ge*_*ij*_ is the G × E effect, *δ*_(*j*)*m*_ is the replicate effect, and *ε*_*ijm*_~N (0, $\sigma_{\varepsilon }^{2}$) is the error term. N stands for normal distribution. To compute BLUEs, *g*_*i*_ was treated as a fixed effect, and the other effects were treated as random effects with each random effect following a specific normal distribution. The model was fitted using the R package lme4 [23].

To calculate broad-sense heritability on an entry-mean basis (*H*^2^), all variables were treated as random effects to estimate their variances using the above model, which was fitted using R package lme4 [23]. The variances of genotype, G × E and error term were identified as $\sigma_{g}^{2},\;\sigma_{ge}^{2}$, and $\sigma_{\varepsilon }^{2}$, respectively. The formula for calculating *H*^2^ is [24]:

$$H^{2}=\frac{\sigma_{g}^{2}}{\sigma_{g}^{2}+\frac{\sigma_{ge}^{2}}{N_{e}}+\frac{\sigma_{\varepsilon }^{2}}{{rN}_{e}}},$$

where *N*_*e*_ is the number of environments, and *r* is the number of replicates.

### Genotyping and data preprocessing

Fresh leaf tissues of the 481 BC_1_F_3_ plants were collected and DNA of each plant was extracted using a cetyltrimethyl ammonium bromide method [25]. DNA samples were sent to CapitalBio Corporation for DNA chip assay, which included 55,000 SNP loci covering the whole genome [26]. The physical position of the SNP markers was based on the B73 RefGen_V3 sequence. SNPs with a calling rate larger than 97% were used. The genotyping data were filtered by removing SNPs with missing data in any parent, SNPs that were non-polymorphic between parents, and SNPs with a missing rate larger than 0.05. Missing markers were imputed with the expected values calculated from estimates of allele frequencies [10], the processed genotypic data were included in S2 File.

### GWAS and selection of large-effect SNPs

GWAS was performed using the R package sommer [27] following the model:

$$y^{*}=X\beta +Zg+W\tau +\varepsilon ,$$

where y* is an N×1 matrix of the BLUEs, **β** is a vector of fixed effects, **g** is the genetic effect, and is treated as a random effect with normal distribution $g∼N(0,K\sigma_{u}^{2}),$ **τ** is the additive marker effects, **ε** is the residual and follows the normal distribution $\varepsilon ∼N(0,I\sigma_{\varepsilon }^{2})$. **X**, **Z**, and **W** are the corresponding design matrixes. **K** was estimated using the A.mat function in the R package rrBLUP with the following formula [5]:

$$K=\frac{WW^{\prime }}{2\sum_{j}p_{j}q_{j}},$$

for the j^th^ marker, p_j_ and q_j_ are the allele frequencies of “A” and “a”, respectively. SNP markers were coded as -1, 0, 1 for the genotypes “aa”, “Aa”, and “AA”, where “aa”, “Aa”, and “AA” were homozygous Zheng58, heterozygous and homozygous PH4CV alleles, respectively. **W** was computed by subtracting **P** from **M** as suggested by VanRaden, where the i^th^ column of **P** is 2(p_i_ − 0.5), **M** is the genotype matrix, and p_i_ is the minor allele frequency of locus i [28].

Considering that flowering time was controlled by a small number of QTL in most biparental populations [2], we selected the top 50 SNPs with the largest -log_10_ (*P*) value to find the SNPs with the largest effects. The 50 SNPS were fitted in a multiple linear model, from which SS_reg_ and SS_tol_ for each SNP were computed. Here, SS_reg_ is the sum of square of each selected SNP, SS_tol_ is the sum of square of the linear model. Phenotypic variance explained (PVE) of each SNP was calculated by dividing SS_reg_ into SS_tol_ [29].

### The effect of fitting large-effect SNPs as fixed effects on the PAs of GS models

The BLUEs were used to test how many large-effect SNPs should be used as fixed effects. PA, calculated as the correlation coefficient between predicted and observed phenotypic data, was obtained by running 100 five-fold cross validations (CVs). The linear mixed model was as follows [30]:

y=Xβ+Zu+ε,

where **y** is the BLUEs, **β** is a matrix containing the fixed effects, **u** is the genetic effect treated as a random effect with $u∼N(0,{K\sigma }_{u}^{2})$, **ε** is the error term with the distribution $\varepsilon ∼N(0,{I\sigma }_{\varepsilon }^{2})$. $\sigma_{u}^{2}$ and $\sigma_{\varepsilon }^{2}$ are the genetic and error variances, respectively. The additive relationship matrix **K** was calculated according to a previous report [31]. **X** and **Z** are the corresponding design matrixes. The above model was fitted using R package BGLR, Gaussian processes (RKHS) model was used for estimating the variances of random effects. The number of iterations and burn-in were set to 20,000 and 5,000, respectively [10].

When the top large-effect SNPs were fitted as fixed effects, **β** included the intercept and the effects of the large-effect SNPs, the genotypic data of the large-effect SNPs were added as columns of the **X** matrix. Meanwhile, the top SNPs were removed from overall markers when calculating **K** matrix [32]. Two-tailed student’s *t*-test analysis was used to test whether fitting one more SNP as fixed effect could increase PA by comparing the 100 PAs calculated by fitting top n SNPs with the 100 PAs calculated by fitting top n-1 SNPs (n≧1).

The above *t*-test analysis revealed that fitting the top four large-effect SNPs as fixed effects was optimal. To test the effect of adding the four large-effect SNPs on PA, four randomly-selected markers were chosen as fixed effects and PA was calculated correspondingly. This process was repeated for 200 times, then the 200 PAs were compared with the PA calculated using the four large-effect SNPs as fixed effects.

To calculate the PA of MAS using the top four large-effect SNPs, the four SNPs was fitted in a multiple regression model using the lm function in R. The phenotype was estimated using the predict function [33]. The PAs were calculated using 100 CVs. In order to prove the effect of MAS using the top four SNPs, we also calculate the PAs of MAS using four randomly-selected SNP.

### GS using three environment models, with and without large-effect SNPs fitted as fixed effects

#### (1) Single environment (SE) model

The SE model can be expressed as:

$$y_{i}={1\mu }_{i}+X\beta_{i}+\varepsilon_{i},$$

where **y**_**i**_ is a vector of phenotypic data in the i^th^ environment, μ_i_ is the overall mean, **β**_**i**_ is a vector of the marker effect, **X** is the genotype matrix, and **ε**_**i**_ is the residual.

#### (2) A-E model

In this model, the marker effect of each SNP in all environments is assumed to be constant, and supposing that we have n environments [16, 34, 35], the model is:

$$\left[\begin{array}{c} y_{1}\\ y_{2}\\ \ldots \\ y_{n} \end{array}\right]=1\left[\begin{array}{c} \mu_{1}\\ \mu_{2}\\ \ldots \\ \mu_{n} \end{array}\right]+\left[\begin{array}{c} X_{1}\\ X_{2}\\ \ldots \\ X_{n} \end{array}\right]\beta +\left[\begin{array}{c} \varepsilon_{1}\\ \varepsilon_{2}\\ \ldots \\ \varepsilon_{n} \end{array}\right],$$

where **y**_**i**_ is the phenotype in the i^th^ (1, 2, …, n) environment, μ_i_ is the overall mean in the i^th^ environment, **X**_**i**_ is the genotype matrix, and **ε**_**i**_ is the residual error.

#### (3) G × E model

In the G × E model, **y**_**i**_ and μ_i_ were the same as those in the A-E model, marker effect **β** was decomposed into two parts, a constant main effect ***β***_**0**_ and the environment-specific effect ***β***_***i***_. The mixed linear model is:

$$\left[\begin{array}{c} y_{1}\\ y_{2}\\ \ldots \\ y_{n} \end{array}\right]=1\left[\begin{array}{c} \mu_{1}\\ \mu_{2}\\ \ldots \\ \mu_{n} \end{array}\right]+\left[\begin{array}{c} X_{1}\\ X_{2}\\ \ldots \\ X_{n} \end{array}\right]\beta_{0}+\left[\begin{array}{cccc} X_{1} & 0 & 0 & 0\\ 0 & X_{2} & 0 & 0\\ 0 & 0 & \ldots & 0\\ 0 & 0 & 0 & X_{n} \end{array}\right]\left[\begin{array}{c} \beta_{1}\\ \beta_{2}\\ \ldots \\ \beta_{n} \end{array}\right]+\left[\begin{array}{c} \varepsilon_{1}\\ \varepsilon_{2}\\ \ldots \\ \varepsilon_{n} \end{array}\right],$$

The three environment models were analyzed in the R package BGLR [10]. The code for implementing A-E and G × E was revised from a previous report [36].

### Cross-validation strategies

The variance components were estimated by fitting the full data set to each of the three models (the SE, A-E, and G × E models). The full data were scaled to standard normal distribution with mean and variance set to zero and one, respectively. In all cases, the number of iterations and burn-in were set to 20,000 and 5,000, respectively.

In the SE analysis, prediction accuracy was calculated using 100 five-fold CVs.

In the multiple environments GS models (the A-E and G × E models), two different CV schemes (CV1 and CV2, S1 Table) were used according to different breeding practices [16, 35, 37]. Briefly, CV1 was designed to predict the performance of newly-developed or untested lines that were not evaluated in any environment. CV2 was designed to predict the phenotype of some materials that was missing or not evaluated in some environments. Because one pair of environments was used to perform multi-environments GS each time, and the number of families in the two environments was different, the CV was performed based on the minimum number of families evaluated in the pair of environments.

PA was calculated as the correlation coefficient between the predicted and observed phenotype for either of the three models.

## Results

### Phenotypic data analysis

**Fig 1:**
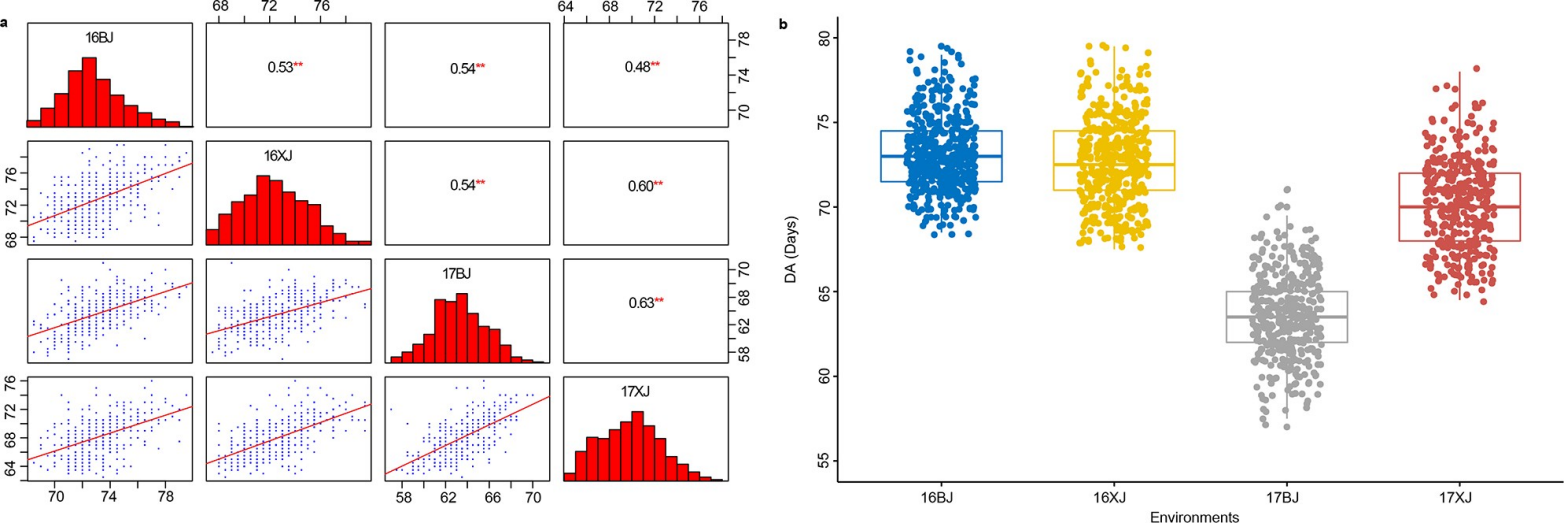
Distribution of days to anthesis (DA) and the correlation of DA between each pair of environments. (a): Distribution of DA evaluated in each of the four environments and their correlation, ** indicates P ≤ 0.01. BJ: Beijing location, XJ: Xinjiang location. The four environments were identified as 16BJ, 16XJ, 17BJ, and 17XJ, respectively; (b): Boxplots showed the distribution of DA evaluated in each environment.

DA of the 481 BC_1_F_3:4_ families were evaluated in four environments over two years (16BJ, 17BJ, 16XJ, and 17XJ), the families flowered earlier in 17BJ (Table 1, Fig 1A and 1B). The correlation coefficients between each pair of environments varied from 0.48 to 0.63, suggesting that DA shared a common genetic basis across all environments (Fig 1A). The heritability estimated across multiple environments and the coefficients of variance proved the stability of DA (Table 1).

**Table 1:** Basic description of days to anthesis (DA) of the BC_1_F_3:4_ population.

Environment^a^	N^b^	Mean±SD^c^	Range	CV (%)^d^	Heritability^e^
16BJ	480	73.09±2.13	68.5–79.5	2.91	0.64
16XJ	467	72.68±2.57	67.5–79.5	3.53	
17BJ	410	63.55±2.43	57.0–71.0	3.83	
17XJ	355	70.13±2.69	64.5–78.0	3.84	

^a^ BJ: Beijing location, XJ: Xinjiang location. The four environments were identified as 16BJ, 16XJ, 17BJ, and 17XJ, respectively.

^b^ N indicates the number of families

^c^ Mean indicates the mean value of DA in the respective environment, SD indicates standard deviation

^d^ CV: coefficient of variance

^e^ Heritability was calculated across all environments

### Genotypic data analysis

**Fig 2:**
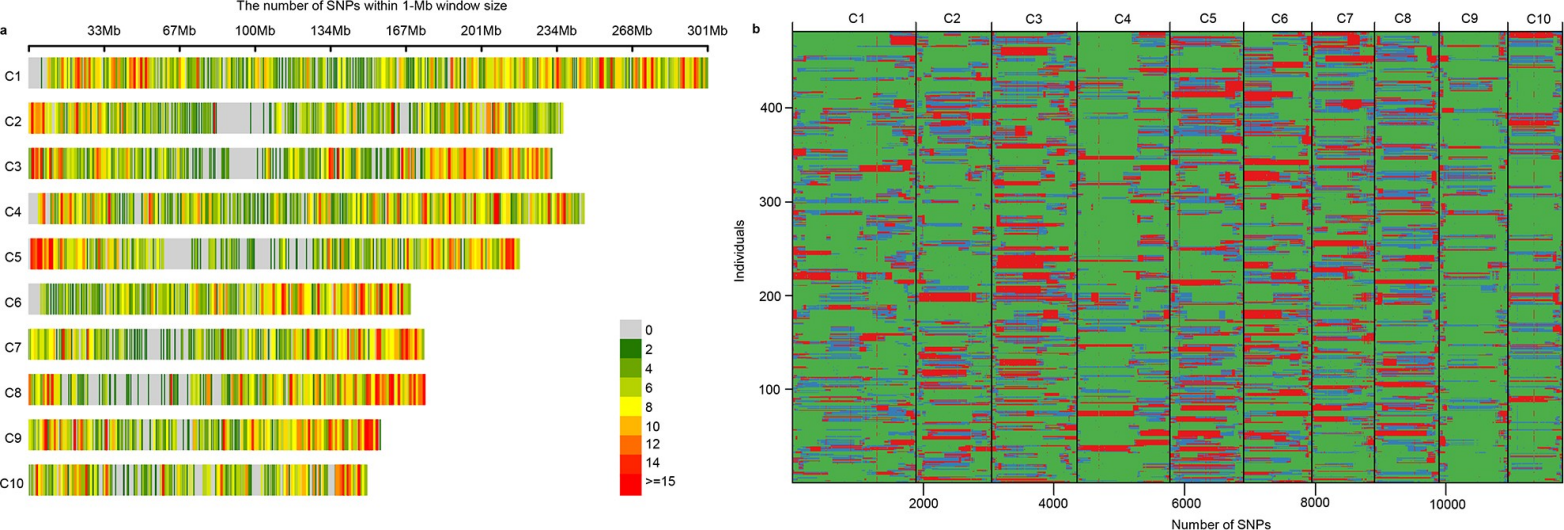
Distribution of 11,781 polymorphic SNPs in the maize genome, and genetic composition of each of the 481 BC_1_F_3_ plants. (a): Heatmap of SNP density on the chromosome within 1-Mb interval, colors were used to indicate the number of SNP within 1-Mb interval. C1, C2, …, C10 represented the ten chromosomes. The physical position of the markers was based on the B73 RefGen_V3 sequence; (b): The genetic background of the 481 BC_1_F_3_ plants. The colors green, red, and blue indicated PH4CV, Heterozygote, and Zheng58 genotypes, respectively.

In total, 11,781 polymorphic SNP markers were obtained after filtering, these markers distributed across the whole genome with a sufficiently high density for GS analysis (Fig 2A). Genotypic analysis of the 481 BC_1_F_3_ plants revealed that the backgrounds of most plants were the homozygous PH4CV genotype, which covered 65.4% of the genome on average. The average coverages of homozygous Zheng58 and heterozygous genotypes were 16.0% and 18.6%, respectively (Fig 2B; S2 Fig; S2 Table). Zheng58 alleles were present across the whole genome, although it was the donor parent (Fig 2B), suggesting that the BC_1_F_3_ population was segregating across the whole genome.

### GWAS and mutiple linear regression analysis identified large-effect SNPs

**Fig 3:**
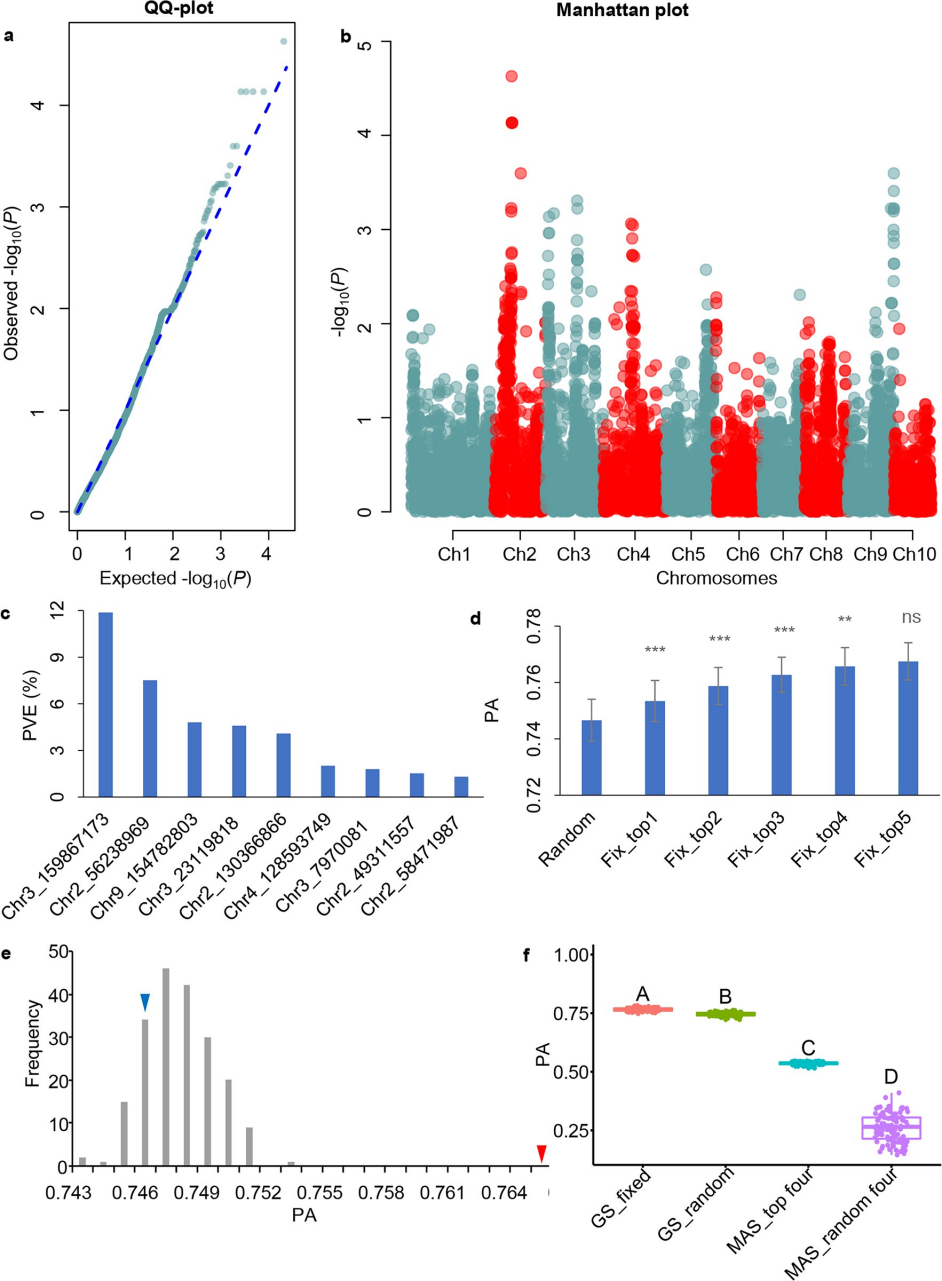
Identification of large-effect SNPs and comparison of the PAs of various models. (a): QQ plot of *P* values; (b): Manhattan plot of GWAS analysis; (c): PVE of the top 50 SNPs were calculate using multiple regression analysis, SNPs with PVE larger than 1% were shown; (d): Student’s *t*-test was performed to compare the PAs of GS models fitting the top n SNPs as fixed effects and the PAs of GS models fitting the top n-1 SNPs as fixed effects (n is an integer ranging from 1 to 4). *** indicated *P* value <0.001, **indicated *P* value <0.01, ns indicated not significant; (e): Frequency distribution of PAs calculated by choosing four randomly-selected SNPs as fixed effects. The process was repeated for 200 times. In each repeat, 100 five-fold cross-validations were performed. Red solid triangle indicated the PA (0.7657) of GS model with the four large-effect SNPs fitted as fixed effects. Blue solid triangle indicated the PA (0.7466) of GS model with no SNP fitted as fixed effect; (f): Comparison of the PAs of GS models and MAS models. GS_fixed indicates the PA of GS model fitting the top four SNPs as fixed effects. GS_random indicates the PA of GS model with all SNPs fitted as random effects. MAS_top four indicates the PA of MAS using the top four SNPs. MAS_random four indicates the PA of MAS using four randomly-selected SNPs. A, B, C, D indicate there are significant differences at 0.01 level.

GWAS was used to identify the genetic basis of DA, QQ plot revealed that the GWAS model was well-fitted in the population under study. Manhattan plot revealed that the highest peak was on chromosome 2, followed by chromosome 9 (Fig 3A and 3B). To identify the loci with large effects, the top 50 SNPs with the largest -log_10_(*P*) values were selected and fitted using a multiple linear regression model, then PVE of each SNP was calculated. Chr3_159867173, an SNP on chromosome 3, had the largest PVE of 11.88%, followed by Chr2_56238969, Chr9_154782803 and Chr3_23119818, explaining 7.52%, 4.81% and 4.59% of total phenotypic variance, respectively (Fig 3C).

### The PA of GS model fitting the top four large-effect SNPs as fixed effects outperform the other models

The BLUEs were used to determine how many large-effect SNPs should be fitted as fixed effects. The PAs of GS models increased with the increase of the number of top SNPs fitted as fixed effects. Student’s *t*-test analysis revealed that the increase of PA was not significant when the number large-effect SNPs increased from four to five (Fig 3D). Therefore, fitting the top four SNPs with the largest effects was optimal. To further demonstrate that the PA increase didn’t happen by chance, four randomly-selected SNPs were fitted as fixed effects using the GBLUP model. The PA calculated by fitting the four large-effect SNPs as fixed effects was larger than each of the 200 PAs of GS models fitting four randomly-selected SNPs as fixed effects (Fig 3E). By comparing the PA of GS models with the PA of MAS, we found that the PA of MAS using the top four SNPs was lower than the PA of GS models. The PA of MAS using the top four SNPs was higher than that of MAS using four randomly-selected SNPs (Fig 3F). The above analysis revealed that the four large-effect SNPs should represent real QTL and could be fitted as fixed effects in the following analysis.

### Fitting the four large-effect SNPs as fixed effects generally decreased genetic variances and increased PA

To investigate the effects of fitting the four large-effect SNPs as fixed effects, the variance components were dissected using the full data. For the SE model, the genetic variances of the GS models decreased when the four large-effect SNPs were fitted as fixed effects (Table 2; S3 Fig). For the A-E and G × E models, the most evident differences were the decreases of the genetic variances when the four large-effect SNPs were fitted as fixed effects. For each of the two G × E interaction variances ($\sigma_{u_{1}}^{2}$ and $\sigma_{u_{2}}^{2}$ in Table 2), no constant differences were found when the four SNPs were fitted as fixed effects (Table 2). The analysis demonstrated that fitting the four large-effect SNPs as fixed effects would generally decrease the genetic variances, and that the four loci had constant effects across the four environments.

**Table 2:** The genetic and error variances of the SE, A-E and G × E models and G × E interaction variances of the G × E model.

	SE Model (Fixed SE Model)^b^				A-E Model (Fixed A-E Model)^b^						G × E Model (Fixed G × E Model)^b^					
Variances^a^	16BJ	16XJ	17BJ	17XJ	16BJ_16XJ	16BJ_17BJ	16BJ_17XJ	16XJ_17BJ	16XJ_17XJ	17BJ_17XJ	16BJ_16XJ	16BJ_17BJ	16BJ_17XJ	16XJ_17BJ	16XJ_17XJ	17BJ_17XJ
$\sigma_{\varepsilon }^{2}$	0.631 (0.657)	0.471 (0.503)	0.406 (0.403)	0.349 (0.430)	0.544 (0.547)	0.524 (0.526)	0.543 (0.541)	0.501 (0.497)	0.452 (0.447)	0.396 (0.407)	0.512 (0.517)	0.489 (0.494)	0.513 (0.513)	0.431 (0.429)	0.422 (0.428)	0.356 (0.368)
$\sigma_{u_{0}}^{2}$	0.368 (0.275)	0.375 (0.178)	0.347 (0.246)	0.481 (0.346)	0.399 (0.283)	0.396 (0.218)	0.369 (0.223)	0.336 (0.226)	0.378 (0.290)	0.442 (0.252)	0.351 (0.233)	0.313 (0.146)	0.245 (0.139)	0.154 (0.097)	0.308 (0.158)	0.386 (0.221)
$\sigma_{u_{1}}^{2}$											0.079 (0.080)	0.107 (0.094)	0.091 (0.085)	0.222 (0.195)	0.062 (0.133)	0.085 (0.062)
$\sigma_{u_{2}}^{2}$											0.070 (0.083)	0.076 (0.070)	0.094 (0.093)	0.130 (0.101)	0.089 (0.080)	0.094 (0.083)

^a^ $\sigma_{\varepsilon }^{2}$: error variance, $\sigma_{u_{0}}^{2}$: genetic variance, $\sigma_{u_{1}}^{2}$ and $\sigma_{u_{2}}^{2}$: the G × E interaction variance with each of the two environments

^b^The fixed SE Model, Fixed A-E Model, and Fixed G × E Model indicated fitting the four large-effect SNPs as fixed effects in the SE Model, A-E Model, and G × E Model, respectively.

The PAs of the three models (the SE, A-E, and G × E models) was calculated to demonstrate the effect of fitting the four large-effect SNPs as fixed effects. For the SE model, it was demonstrated that the PA increased when the four large-effect SNPs were fitted as fixed effects for each environment (S3 Fig). We also found that fitting the top four SNP identified in each environment as fixed effects could also increase PA (S4 Fig). For the multi-environment GS model including A-E model and G × E model, two cross-validation (CV) schemes, named as CV1 and CV2, were used (S1 Table). For the A-E model, fixing the four SNPs generally resulted in higher PAs for the CV1 and CV2 schemes (excluding one case in CV1 and three cases in CV2, Table 3). The results were similar for the G × E model. Generally speaking, the results suggested that fitting the four large-effect SNPs as fixed effects was advisable for each of the three models.

**Table 3:** Fitting the four large-effect SNPs as fixed effects and a G × E component generally enhanced genomic prediction.

Pairs of environments	Correlation^a^	CV1				CV2			
		A-E model^b^	Fixed A-E model^b^	G × E model^b^	Fixed G × E model^b^	A-E model^b^	Fixed A-E model^b^	G* × E model^b^	Fixed G × *E model^b^
16BJ_16XJ	0.530	0.523	0.538	0.527	0.541√	0.567	0.577	0.573	0.578√
		0.630	0.633	0.640	0.647√	0.666√	0.665	0.665	0.665
16BJ_17BJ	0.544	0.513	0.534√	0.509	0.530	0.545	0.553√	0.534	0.546
		0.687	0.706	0.701	0.716√	0.730	0.738	0.731	0.742√
16BJ_17XJ	0.481	0.508	0.528√	0.512	0.528√	0.507	0.521√	0.506	0.519
		0.698	0.698	0.714	0.722√	0.714	0.715	0.724	0.731√
16XJ_17BJ	0.538	0.614	0.615	0.629	0.632√	0.629	0.626	0.653	0.654√
		0.658	0.680	0.699	0.715√	0.681	0.703	0.723	0.739√
16XJ_17XJ	0.604	0.649	0.650√	0.645	0.647	0.679√	0.677	0.669	0.667
		0.708	0.723	0.720	0.734√	0.734	0.740	0.744	0.750√
17BJ_17XJ	0.627	0.681	0.704	0.688	0.709√	0.716	0.727	0.722	0.732√
		0.699	0.709	0.715	0.727√	0.728	0.725	0.739	0.740√

^a^Correlation coefficients between days to anthesis of each pair of environments

^b^Each pair of environments corresponded to two PAs. The phenotype data of the two environments was used as the training set, the first and second PA was calculated by treating the phenotype data of the first and second environments as the validation sets, respectively. The fixed A-E model and fixed G × E model indicated fitting the four large-effect SNPs as fixed effects in the A-E Model and G × E Models, respectively.

√ indicated the largest PA among the four values in the same row

### The G × E models with the four large-effect SNPs fitted as fixed effects generally had better performance

When comparing the two multi-environment models (the A-E and G × E models) without fitting any SNP as a fixed effect, the G × E models had better performance than the A-E models in ten of the twelve cases for the CV1 scheme, and in eight of the twelve cases for the CV2 scheme (Table 3). When comparing the two multi-environment models with the four large-effect SNPs fitted as fixed effects, the G × E models outperformed the A-E models in nine of the twelve cases for the CV1 scheme and in eight of the twelve cases for the CV2 scheme (Table 3). The results supported that the PAs of the G × E models were generally larger than those of the A-E models.

Because both fitting large-effect SNPs as fixed effects and modeling G × E interaction could increase PA, it was assumed that the best prediction could be achieved using the models including both of the two factors. By looking through each row in Table 3, it could be found that the G × E models fitting the four large-effect SNPs as fixed effects had the highest PAs in ten of the twelve cases for the CV1 scheme and in eight of the twelve cases for the CV2 scheme. Therefore, including the two factors into the GS models should be a powerful strategy for enhancing GS efficiency.

## Discussion

With the fast development of genome sequencing technology and the continual decreasing of the genotyping cost, efficient selection is becoming increasingly important for any commercial breeding programme. GS can increase breeding efficiency by making prediction at the seedling stage as soon as DNA of the prediction population was available, thus help breeders to exclude undesired genotypes. GS could also increase breeding efficiency by making the best prediction and selecting the desired plants at the decision-making stage of a GS breeding programme. This study was designed to examine how to make the best use of available genotypic and phenotypic data to make the best prediction by including additional components to the GS models. The analysis in this study suggested that, compared with the use of crude GS models, manipulating existing data using statistical approaches enhanced genomic prediction without increasing any cost.

The finding that using known genetic loci as fixed effects could increase PA highlighted the importance of obtaining and assessing these data [14, 38]. There are two general approaches to obtaining these data: summarizing the chromosome position of QTL and genes by retrieving published articles; performing QTL analysis using established training population with both genotypic and phenotypic data. It should be noted that the collected historical QTL and gene information might not be useful if not validated in the breeding population. However, even when QTL and gene information are validated in the training population, this information should be carefully examined. QTL of a specific trait can be influenced by heritability and genetic architecture, the target trait might be controlled by one or two major QTL, or by many minor-effect QTL. It was demonstrated using simulation data that the selection efficiency increased with the increase of heritability for a given genetic architecture where only one locus with a major effect was fitted as a fixed effect. The increase in prediction accuracy was negligible when the effect of a locus fitted as fixed effect was 5% [13]. However, in this study, we proved using real data that the increase in prediction accuracy was significant even the effects of three loci were less than 10% (Fig 3D), which might be related to the relatively high heritability of DA in this study.

Our analysis showed that fitting large-effect SNPs as fixed effects enhance GS. The prerequisite is that each of the large-effect SNPs should be in linkage disequilibrium with a real QTL. The four SNPs were in the chromosome regions of maize bin 3.05, 2.04, 9.07 and 3.04 according to ISU Integrated IBM 2009 (https://www.maizegdb.org/data_center/map). These regions contained consensus QTL according to QTL meta-analysis [2, 39], suggesting that the four SNPs detected in this study should represent real QTL.

Marker effects estimated using mixed models might not reflect the real genetic effects, because the genetic variance of each SNP was assumed to follow some prior distribution, and modeling of this prior distribution might affect the estimation of marker effects, especially for large-effect SNPs [10, 13, 40]. When markers with large effects were fitted as fixed effects, only the effects of the remaining SNP markers should be estimated, strong shrinkage could be avoided in estimating the effects of large-effect SNPs when solving GS models [5, 10]. Thus, the GEBVs can be estimated accurately when major genes are fitted as fixed effects.

In maize breeding programmes, a frequently-used strategy is to select lines from the advanced generation formed by crossing two elite inbred lines. However, GS studies modeling large-effect SNPs as fixed effects and G × E interaction effects using this kind of breeding population are relatively few. Therefore, it is necessary to examine how the PAs of GS models would change when the two factors are included in the GS models using a maize biparental population. Our study was conducted to address this concern, and we ultimately proved that fitting large-effect SNPs as fixed effects in the GS models would increase PA in a maize biparental population, even the effects of some SNPs were less than 5%. Furthermore, GS models fitting large-effect SNPs as fixed effects and G × E effects generally had the best performance. Our results should be useful for molecular crop breeding.

## Conclusion

GWAS and multiple linear regression analysis was successfully applied to identify large-effect SNPs. Using the BLUEs, it was demonstrated that fitting the four large-effect SNPs as fixed effects increased PA and decrease genetic variance. We further demonstrated that combining G × E interaction and fitting large-effect SNPs as fixed effects could generally increase PA.

## Supporting information

S1 File Phenotypic data. DA of the BC_1_F_3:4_ population composed of 481 families was evaluated in four environments, including 16BJ, 16XJ, 17BJ, and 17XJ.
(ZIP)

S2 File Genotypic data. In the data file, 1, 0, and -1 represented homozygous PH4CV, heterozygous, and homozygous Zheng58 genotypes, respectively.
(ZIP)

S1 Fig Flowchart for the construction of the 481 BC_1_F_3:4_ families used in this study. (TIF)

S2 Fig Frequency distribution of the three genotypes in the 481 BC_1_F_3_ plants. For each BC_1_F_3_ plant, the proportion of each of the three genotypes (Zheng58 homozygous, heterozygous, and PH4CV homozygous) was calculated, generating 481 values for each of the three genotypes. The distribution was plotted using the data of the three genotypes with each containing 481 values.
(TIF)

S3 Fig Fitting the four large-effect SNPs as fixed effects could decrease genetic variance and increase PA for each environment. The genetic variance (a) and residual variance (b) for each environment were dissected with or without the four large-effect SNPs as fixed effects. PA was also calculated for each environment with or without the four large-effect SNPs as fixed effects (c). Fixed and Random indicated fitting the four large-effect SNPs as fixed effects and four randomly-selected SNP as fixed effects, respectively. *** on top of gray column indicated significantly different from its left column at *P* < 0.001 level. *P* value were determined by two-tailed Student’s *t*-test.
(TIF)

S4 Fig Fitting the four SNPs identified in each environment as fixed effects could increase PA. GWAS was performed using the phenotypic data in each environment and the top four SNPs were identified accordingly. For each environment, PA was calculated with or without the top four SNPs as fixed effects. Fixed and Random indicated fitting the top four SNPs as fixed effects and four randomly-selected SNPs as fixed effects, respectively. *** indicated significantly different at *P* < 0.001 level. *P* value were determined by two-tailed Student’s *t*-test.
(TIF)

S1 Table The two cross validation schemes adopted to test the PA of AE and G*E GS models. CV1 and CV2 mean two cross validation schemes, Env1, Env2, ……, Envn means there are n environments, NA means phenotype was not evaluated in the specific environment, N means there are N lines.
(DOCX)

S2 Table The proportion of each of the three genotypes in the 481 BC_1_F_3_ plants. This table was the summarized according to S2 Fig. A total of 481 values were calculated for each of the three genotypes, the mean, minimum, and maximum values were derived from the 481 values.
(DOCX)

## Abbreviations

GS: Genomic Selection
PA: Predication Accuracy
SNP: Single Nucleotide Polymorphism
G × E: Genotype-by-Environment
QTL: Quantitative Trait Locus/Loci
MAS: Marker-Assisted Selection
GEBVs: Genomic Estimated Breeding Values
rrBLUP: ridge regression Best Linear Unbiased Prediction
GBLUP: Genomic Best Linear Unbiased Prediction
GWAS: Genome Wide Association Study
DA: Days to Anthesis
BLUE: Best Linear Unbiased Estimate
ANOVA: Analysis of Variance
PVE: Phenotypic Variance Explained
CV: Cross-Validation
SE: Single Environment
A-E: Across-Environment
