# Correction: Enhancing genomic selection by fitting large-effect SNPs as fixed effects and a genotype-by-environment effect using a maize BC_1_F_3:4_population

**DOI:** 10.1371/journal.pone.0226592

**Published:** 2019-12-12

**Authors:** Dongdong Li, Zhenxiang Xu, Riliang Gu, Pingxi Wang, Demar Lyle, Jialiang Xu, Hongwei Zhang, Guoying Wang

The eighth author’s name is spelled incorrectly. The correct name is: Guoying Wang.
