# Supplementary figures and images for: Enhancing genomic selection by fitting large-effect SNPs as fixed effects and a genotype-by-environment effect using a maize BC1F3:4 population
Source: PLoS One. 2019 Oct 17;14(10):e0223898. doi: 10.1371/journal.pone.0223898 (PMC6797203; doi:10.1371/journal.pone.0223898)

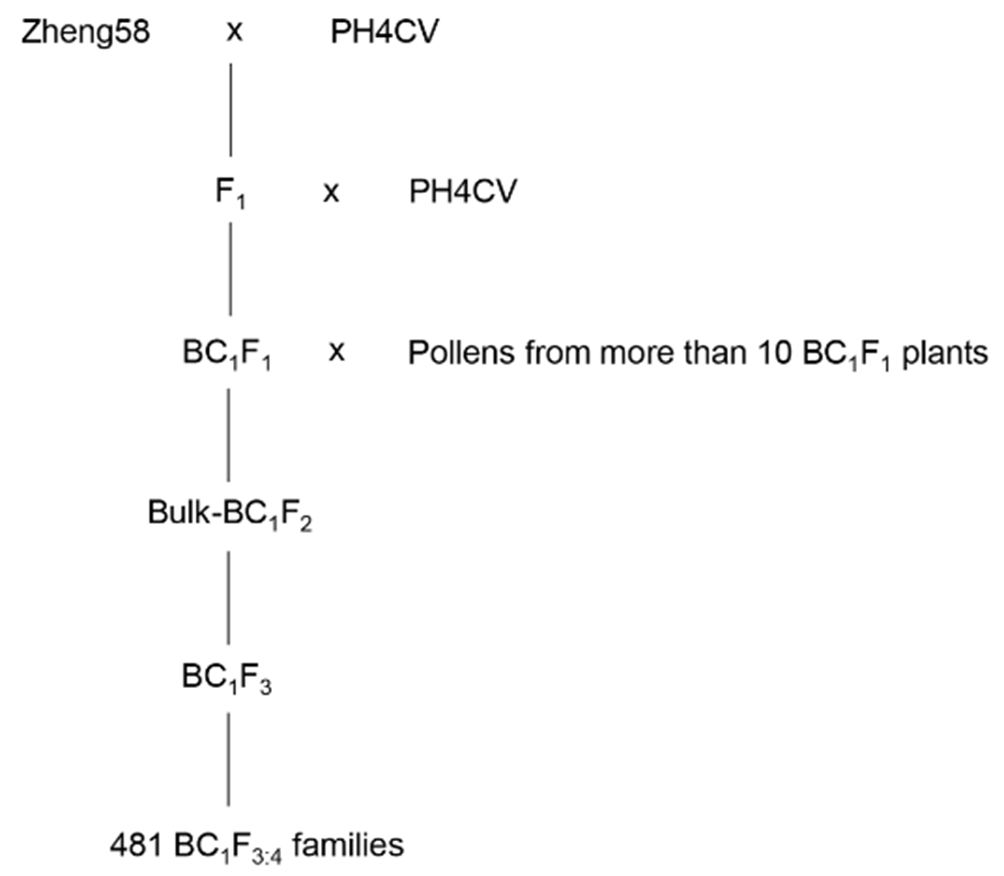

Supplement: S1 Fig — (TIF) [file pone.0223898.s003.tif]

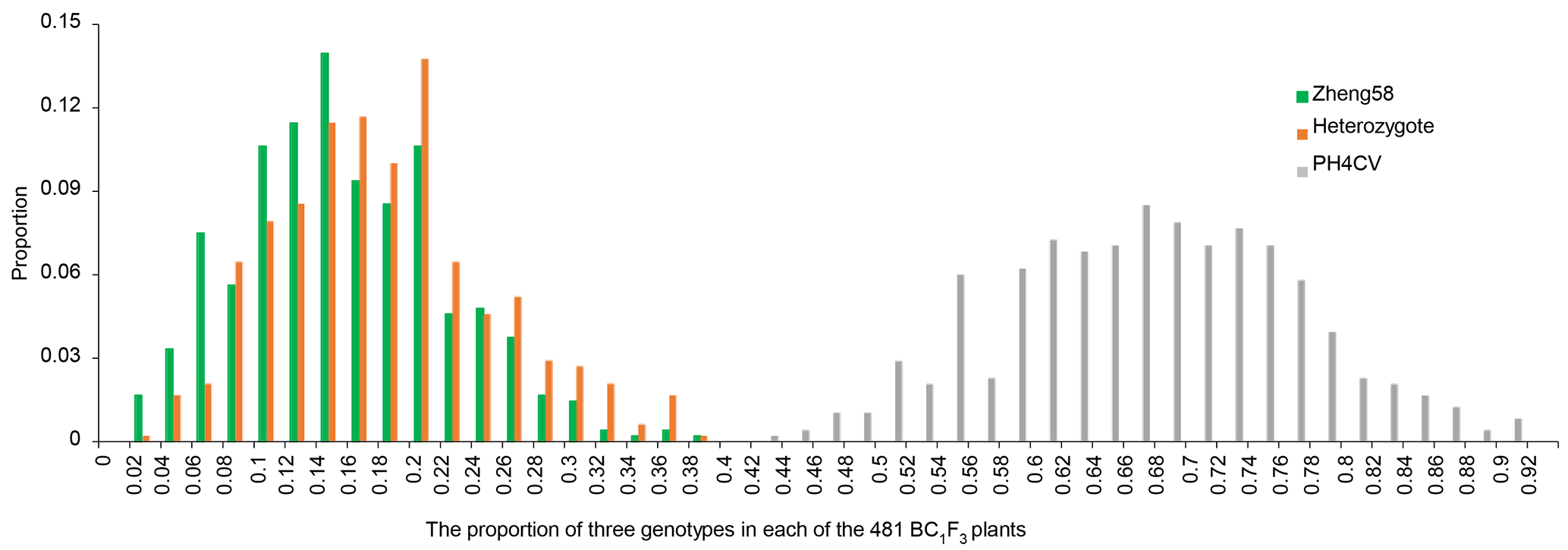

Supplement: S2 Fig — For each BC1F3 plant, the proportion of each of the three genotypes (Zheng58 homozygous, heterozygous, and PH4CV homozygous) was calculated, generating 481 values for each of the three genotypes. The distribution was plotted using the data of the three genotypes with each containing 481 values. (TIF) [file pone.0223898.s004.tif]

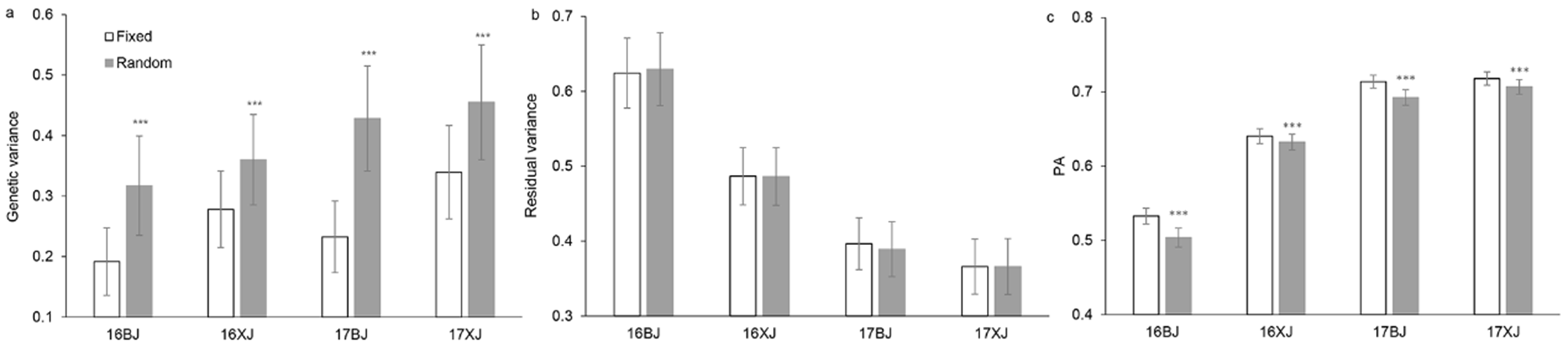

Supplement: S3 Fig — The genetic variance (a) and residual variance (b) for each environment were dissected with or without the four large-effect SNPs as fixed effects. PA was also calculated for each environment with or without the four large-effect SNPs as fixed effects (c). Fixed and Random indicated fitting the four large-effect SNPs as fixed effects and four randomly-selected SNP as fixed effects, respectively. *** on top of gray column indicated significantly different from its left column at P < 0.001 level. P value were determined by two-tailed Student’s t-test. (TIF) [file pone.0223898.s005.tif]

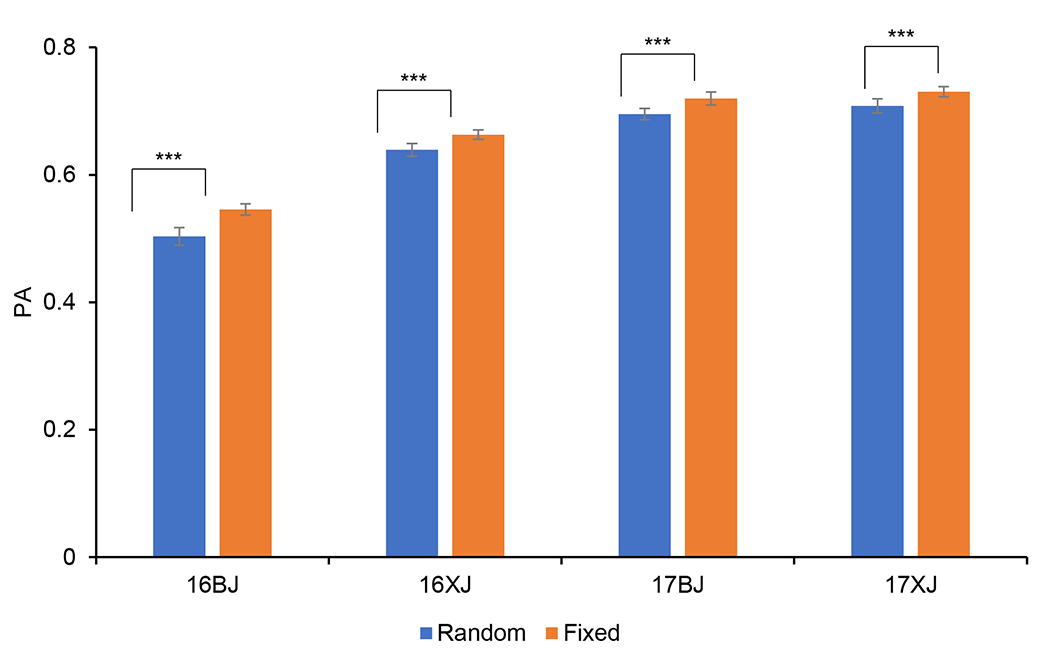

Supplement: S4 Fig — GWAS was performed using the phenotypic data in each environment and the top four SNPs were identified accordingly. For each environment, PA was calculated with or without the top four SNPs as fixed effects. Fixed and Random indicated fitting the top four SNPs as fixed effects and four randomly-selected SNPs as fixed effects, respectively. *** indicated significantly different at P < 0.001 level. P value were determined by two-tailed Student’s t-test. (TIF) [file pone.0223898.s006.tif]
