# Supplementary material for: Enhancing genomic selection by fitting large-effect SNPs as fixed effects and a genotype-by-environment effect using a maize BC1F3:4 population
Source: PLoS One. 2019 Oct 17;14(10):e0223898. doi: 10.1371/journal.pone.0223898 (PMC6797203; doi:10.1371/journal.pone.0223898)
Supplement: S1 Table — CV1 and CV2 mean two cross validation schemes, Env1, Env2, ……, Envn means there are n environments, NA means phenotype was not evaluated in the specific environment, N means there are N lines. (DOCX) [file pone.0223898.s007.docx]

**S1 Table The two cross validation schemes adopted to test the PA of AE and G*E GS models**

|  | CV1 | | | | CV2 | | | |
| --- | --- | --- | --- | --- | --- | --- | --- | --- |
| LINE | Env1 | Env2 | … | Envn | Env1 | Env2 | … | Envn |
| Line1 | y1 | y1 | … | y1 | y1 | y1 | … | NA |
| Line2 | y1 | y1 | … | y1 | y2 | y2 | … | y2 |
| Line3 | NA | NA | … | NA | NA | NA | … | y3 |
| Line4 | NA | NA | … | NA | y4 | y4 | … | NA |
| Line5 | y5 | y5 | … | y5 | NA | NA | … | y5 |
| … | … | … | … | … | … | … | … | … |
| Line90 | y90 | y90 | … | y90 | y90 | y90 | … | NA |
| Line91 | NA | NA | … | NA | NA | NA | … | y91 |
| Line92 | y92 | y92 | … | y92 | y92 | y92 | … | NA |
| Line93 | y92 | y92 | … | y92 | y93 | y93 | … | y93 |
| Line94 | y92 | y92 | … | y92 | NA | NA | … | y94 |
| Line95 | NA | NA | … | NA | y95 | y95 | … | y95 |
| … | … | … | … | … | … | … | … | … |
| LineN | yN | yN | … | yN | yN | yN | … | yN |

CV1 and CV2 mean two cross validation schemes, Env1, Env2, ……, Envn means there are n environments, NA means phenotype was not evaluated in the specific environment, N means there are N lines
