# Supplementary material for: Enhancing genomic selection by fitting large-effect SNPs as fixed effects and a genotype-by-environment effect using a maize BC1F3:4 population
Source: PLoS One. 2019 Oct 17;14(10):e0223898. doi: 10.1371/journal.pone.0223898 (PMC6797203; doi:10.1371/journal.pone.0223898)
Supplement: S2 Table — This table was the summarized according to S2 Fig. A total of 481 values were calculated for each of the three genotypes, the mean, minimum, and maximum values were derived from the 481 values. (DOCX) [file pone.0223898.s008.docx]

**S2 Table The proportion of each of the three genotypes in the 481 BC_1_F_3_ plants**

|  | Mean | Minimum | Maximum |
| --- | --- | --- | --- |
| Zheng58 genotype | 16.0% | 2.1% | 38.2% |
| Heterozygous | 18.6% | 3.7% | 38.4% |
| PH4CV genotype | 65.4% | 41.4% | 88.9% |

This table was the summarized according to Figure S2. A total of 481 values were calculated for each of the three genotypes, the mean, minimum, and maximum values were derived from the 481 values
